# The Engineering Side of Hospital Work

**Published:** 1905-03-11

**Authors:** 


### HOSPITAL ADMINISTRATION.

### CONSTRUCTION AND ECONOMICS.

### THE ENGINEERING SIDE OF HOSPITAL WORK.

*Continued from page 376.*

VI.—REFUSE, DESTRUCTORS, OR INCINERATORS.

The apparatus designed for the complete destruction of refuse and other matter for hospitals, has been worked out on two lines, both of which, from the reports given to the writer, answer very well. In one form there is a combustion chamber, with a door for freeing cinders, etc., in front of the apparatus, and a second door on the top, into which the refuse is delivered. The door on the top is made large enough to take mattresses, and similar objects and is sometimes fitted with a hopper. The combustion chamber is something on the lines of the ordinary boiler furnace, with fire-bars, upon which combustion takes place, and an ash-pit into which the unconsumed matter falls. And the draught is what is termed natural, that obtained from the chimney, into which the hot gases formed by the combustion pass. In addition to the combustion chamber proper, there is a subsidiary combustion chamber, at the back of the main combustion chamber, between it and the chimney, in which coal or coke is burnt, on fire-bars in the usual way, the office of the secondary chamber being to cremate any of the organic compounds that may come over. This form of apparatus is in use at the Middlesex and London Hospitals, and by the reports of the engineers of both hospitals, answered its purpose very well indeed. The writer was assured that there was never any smell from it, nor had there been any nuisance of any kind. At Middlesex the steam and hot air from the disinfector are discharged over the burning refuse, so that there should be no chance of nuisance from that cause, the steam and air, and any bacilli they carried being thoroughly cremated in the combustion chamber. The writer understands that the furnace mentioned, which is made by Messrs. Manlove, Alliot, and Co., of Nottingham, takes everything coming from the operating theatre, the post-mortem room, etc., without any trouble, and without smell. It will be seen that the arrangement is a very simple one, and that it is completely under the control of the attendant, who can make up his secondary fire to what he pleases, so that any heat that may be necessary can be obtained.

#### Refuse Destructors with Forced Draught.

The other line that has been followed in hospital refuse destructors has been the suppression of the secondary fire, and the production of increased heat by means of a special arrangement for adding to the supply of air passing to the furnace. There are two varieties of this arrangement on the market. In one, that made by Messrs. Meldrum Bros., of Manchester, which has been fitted up for several of the hospitals of the Metropolitan Asylums Board, the whole of the operations take place within the one chamber, the matter to be destroyed being put into a hopper in the front of the furnace, and dried before it passes on to the fire-bars; any green gases, or noxious fumes that are emitted by the refuse, it is claimed, being thoroughly cremated by having to pass over the glowing mass in the combustion-chamber. Between the combustion-chamber proper, and the drying-hearth, a fire-brick arch is built, which becomes very hot, and assists to dry the newly-delivered refuse, as well as assisting to cremate any organisms, and to break up any organic compounds. The main combustion-chamber, which is of the usual pattern with fire-bars, upon which the material to be consumed rests, is kept at a very high temperature, 2,000° F., it is claimed, so that the organic compounds are thoroughly broken up before the products of combustion pass into the chimney. The combustion-chamber is made large, and this, it is stated, provides for the point mentioned in the previous article, the settling of the dust. In hospital refuse there is naturally less dust than in town's refuse, and in both patterns of apparatus it is easily stopped in the combustion chamber. In Messrs. Meldrum's apparatus the air passing into the furnace is not heated, though there would be no difficulty in arranging that it should be. The high temperature obtained in the combustion chamber is due entirely to the action of the forced draught, which it may be as well to explain here.

#### Forced Draught.

Forced draught is the modern engineer's method of making his furnace, whatever it may be consuming, work at a higher rate without increasing the size of his chimney. Combustion, it will be remembered, is the chemical combination of carbon and other elements with oxygen, presented to them in the form of atmospheric air. When carbon combines with oxygen the gases that are produced by the combination are able to exist with less energy than the oxygen gas to which they owe their being, and hence heat is liberated. When 1 lb. of carbon combines with a certain quantity of oxygen, in the proportion of 1 atom of carbon to 1 atom of oxygen, forming carbonic oxide, approximately 10,000 B.Th. units are liberated, and when the carbonic oxide takes up a second atom of oxygen a further 4,000 B.Th. units are liberated. The ability to combine with oxygen naturally depends upon the presence of the required quantity of oxygen, and this again, under the conditions ruling where combustion takes place, depends upon the presence of a certain quantity of air. Air as we know is composed of a mixture of oxygen and nitrogen in the proportion, approximately, of 1 to 4, together with other gases, the vapour of water, and other substances that do not concern the subject under discussion. In the old days the air for any fire was provided by the draught created by the chimney, and there was no means of controlling the supply of air except by partially closing the damper. You could lessen the supply of air, you could not add to it, without adding to the height of your chimney. The draught created by the chimney was due to the difference in weight between the column of hot gases in the chimney and the equivalent column of air outside. As the chimney aged it usually became less efficient because it became partially filled up. The modern engineer has changed all that by the use of what has been termed forced draught. The term, like so many others in engineering, is not a very good one, as force is not used any more than with a chimney. The weight of a column of air is just as forcible, if there is enough of it, as any of the modern methods. The modern methods are twofold. Fans are employed to either force air into the furnace through the fire-bars, the air passing into the ashpit, and thence to the fire, the ashpits being enclosed for the purpose, or by sucking the air through the fire and the ashpit by placing fans on the opposite side of the furnace, between it and the chimney. The other method, which is the one that has been employed in connection with hospital refuse destructors, by those firms who have adopted forced draught, is the use of a jet of steam, passing into the furnace, and causing a lower pressure behind it, into which the air enters. In the Meldrum furnace, the firm's blowers are used. A pipe, connected with the steam supply, is led into the furnace, and steam is caused to pass from the pipe, under the furnace bars, and up through them into the fuel. The steam first condenses on the furnace bars, and is immediately re-evaporated to steam, the process aiding the general arrangement very considerably. The condensation, and re-evaporation tends to keep the furnace bars cool, by the extraction of the heat necessary for evaporation, and the steam passing on to the hot portion of the fire is decomposed into its constituents, hydrogen and oxygen, the recombination of the constituent gases aiding the combustion of the refuse very considerably. Meanwhile the constant passage of the jet of steam through the matter on the fire-bars, causes a constant stream of air to pass through it, with additional combustion. Up to a certain point, this additional quantity of air passing through the refuse, and other substances adds to the chemical actions going on, and to the heat liberated, with the result that much higher temperatures are obtained than are possible with ordinary chimney draught. It should be noted that the principal qualification possessed by so called forced draught is the ability it gives the engineer to regulate his combustion. As was pointed out in the previous article, some of the earlier refuse destructors were nuisances because the dust that was formed was whirled out at the chimney top. With forced draught the engineer can arrange to regulate the quantity of air passing according to the material that he is burning, a very important matter with refuse. In the other apparatus in which forced draught is employed, Messrs. Horsfall's of Leeds, which has been fitted up at the Radcliffe Infirmary at Oxford, steam jets are again used, for creating draught, and for the other purposes. The air passing into the furnace is also warmed on its way, by passing it through heating boxes at the side of the furnace. The steam jets cause a draught of air, but the only way the air can enter is through the side boxes where it is exposed to the heat of the furnace, and from whence it passes, with the steam, to the fire-bars, through the refuse, etc., upon the fire-bars, and thence to the ashpit. Messrs. Horsfall combine their refuse destructor, at the Radcliffe Infirmary, with one of the boilers that is generating steam for the hospital, and in this respect the arrangement differs from that of Messrs. Meldrum, as well as by the use of the air-heating boxes. Beyond the combustion chamber, is a flue, leading to the flues of the boiler. The junction flue is composed of special firebrick, and is kept at a temperature of 2,000° Fahr., thus forming the cremating chamber for the organic compounds. The hot gases generated by the combustion of the refuse pass into and through the boiler flues, heating the water in the boiler, in the usual way, and thence pass up the boiler chimney.

It should perhaps be mentioned that the combustion of hospital refuse, including that coming from the operating theatre, is very much aided by the fact that a great many cinders and similar refuse is made in hospital work. All hospitals have some fires for warming the wards or other rooms, many of them have a good many fires, and there is always a good deal of what engineers who are engaged in refuse destructor work would call good stuff, calorifically speaking. The cinders, small coal, and other refuse are sufficient for all purposes, and it is very rarely that any addition has to be made for the purpose of obtaining additional heat. Where anthracite coal is burnt, a very small quantity of it among the refuse will go a very long way.

Other Refuse Destructors in the London Hospitals.

Of the 12 great London hospitals visited by the writer, St. George's, Charing Cross, Westminster, St. Thomas's, St. Mary's, King's, St. Bartholomew's, and the Royal Free Hospital have no refuse destructors. In some, the boiler furnaces he was told, are used where necessary, in others the refuse is handed over to the local authority. The two, Middlesex and London, using Manlove and Alliott's apparatus have been mentioned. Only two others of the 12 have any refuse destructor, Guy's and University. At Guy's there is an apparatus designed by Mr. Kirkland, one of the consulting engineers of the hospital. The apparatus is very interesting as one of the few about any of the London hospitals visited by the writer where any attempt had been made to utilise the heat given off for the purpose of economising coal. The apparatus is something on the lines of some of the town's apparatus. The refuse is delivered on to a tipping floor, and is fed to the destructor through holes provided for it. On passing through the holes it comes on to a dead plate, where it dries, and is then pushed back on to the fire-bars behind, where it is consumed. The gases and other products of combustion pass into a long horizontal flue, kept at a high temperature, where the organic compounds are broken up, and the hot gases then pass into a Green's Economiser, where they assist to heat the water for the boilers, a part of the heat left in the gases being given to water passing from the cold water tank, through pipes arranged for the purpose on its way to the boiler. The walls of the destructor are hollow and the air for the furnace is warmed by passing through the hollow spaces on its way to the fire-bars. A by pass is arranged in connection with the economiser, so that the gases can pass directly to the chimney, if anything is wrong with the economiser. Economisers will be described later on. At University there is a simple arrangement, consisting of a brick furnace with fire-bars of the ordinary type, and fire-doors in front, through which the refuse is fed on to the fire-bars. There is a steam jet supplied with steam from the boilers under the fire-bars creating a draught, and keeping the bars cool, as already explained, but there appears to be no effort to create any special conditions, beyond those created by the forced draught of the steam jet. In the next article the writer will describe the disinfecting apparatus in use.

Referring to the fact that so many of the London hospitals have no means of destroying quickly and efficiently the refuse that in hospitals, of all places in the world, must be constantly making, it appears to him that if refuse destructors have any value, if they are necessary for towns, as educated opinion is more and more declaring, then their absence in hospitals can only be described as a great danger to the hospitals and to the adjacent communities.

